# A haplotype-resolved chromosomal reference genome for the porcini mushroom *Boletus edulis*

**DOI:** 10.1093/g3journal/jkaf069

**Published:** 2025-04-24

**Authors:** Etienne Brejon Lamartinière, Keaton Tremble, Bryn T M Dentinger, Kanchon K Dasmahapatra, Joseph I Hoffman

**Affiliations:** Faculty of Biology, Department of Evolutionary Population Genetics, Bielefeld University, 33501 Bielefeld, Germany; Department of Biology, Duke University, Durham, NC 27707, USA; School of Biological Sciences, The University of Utah, Salt Lake City, UT 84112, USA; Natural History Museum of Utah, Salt Lake City, UT 84108, USA; Department of Biology, University of York, Heslington, York YO10 5DD, UK; Faculty of Biology, Department of Evolutionary Population Genetics, Bielefeld University, 33501 Bielefeld, Germany; Faculty of Biology, Center for Biotechnology (CeBiTec), Bielefeld University, 33615 Bielefeld, Germany; Joint Institute for Individualisation in a Changing Environment, Bielefeld University and University of Münster, 33501 Bielefeld, Germany; British Antarctic Survey, UKRI-NERC, Cambridge CB3 OET, UK

**Keywords:** *Boletus edulis*, ectomycorrhizal fungi (EMF), reference genome assembly, Hi-C, population structure

## Abstract

Haplotype-resolved chromosomal reference genomes are increasingly available for many fungi, offering insights into the evolution of pathogenic and symbiotic lifestyles. However, these resources remain scarce for ectomycorrhizal fungi, which play crucial roles in forest ecosystems. Here, we used a combination of chromatin conformation capture and PacBio sequencing to construct a haplotype-resolved chromosomal genome assembly for *Boletus edulis*, a prized edible fungus and emerging model for ectomycorrhizal fungal research. Our new reference assembly, “BolEdBiel_h2,” derives from a *B. edulis* sporocarp sampled in Bielefeld, Germany. The genome assembly spans 41.8 Mb, with a scaffold N50 of 4.1 Mb, and includes 11 chromosome-level scaffolds, achieving near telomere-to-telomere coverage across multiple chromosomes. We annotated a total of 15,406 genes, with a Benchmarking Universal Single-Copy Orthologs score of 96.2%. Key genomic features such as mating loci, carbohydrate-active enzymes, and effector proteins, were identified. As a first application of this new genomic resource, we mapped whole-genome resequencing data from 53 genets to investigate the population structure and genetic diversity of the European lineage of *B. edulis*. We identified 2 distinct genetic clusters and found that high-latitude populations from Iceland and Fennoscandia exhibited greater nucleotide diversity than populations from the United Kingdom and Central Europe. Additionally, we discovered a 0.4-Mb inversion on chromosome 3 and identified several regions of locally elevated nucleotide diversity, which may represent candidates for ecological adaptation. This genomic resource will facilitate a deeper understanding of this ecologically and commercially important wild fungus.

## Introduction

Ectomycorrhizal fungi (EMF) play a crucial role in the functioning of forest ecosystems worldwide. They facilitate nutrient cycling (Read and Perez-Moreno 2003), contribute to carbon sequestration (Anthony *et al.* 2024), and enhance the growth, immunity, and pathogen resistance of nearly 60% of all trees (Steidinger *et al.* 2019). However, despite their critical roles in forest ecosystems, our understanding of the ecology and evolution of EMF remains limited. This knowledge gap arises partly from the difficulty of studying these predominantly subterranean organisms, which typically cannot be cultured in the laboratory beyond the mycelium stage due to their symbiotic lifestyles.

Over the past 3 decades, molecular genetic approaches have been pivotal in expanding our understanding of EMF, facilitating both ecological and evolutionary research (Douhan *et al.* 2011). Classical genetic markers such as randomly amplified polymorphic DNAs, amplified fragment length polymorphisms, and simple sequence repeats allowed researchers to distinguish individual genotypes in natural settings, paving the way for studies of clonality and genetic diversity (Bonello *et al.* 1998; Amend *et al.* 2009). More recently, advancements in high-throughput sequencing technologies have broadened the scope of EMF research, providing detailed insights into population structure (Branco *et al.* 2016), gene flow (Tremble, Hoffman, *et al.* 2023), gene content (Kohler *et al.* 2015), adaptation (Bazzicalupo *et al.* 2020), and molecular evolution (Looney *et al.* 2022).

A key requirement for modern population genomic studies is the availability of high-quality, annotated, chromosome-level reference genomes. These resources enable gene discovery (Mariene and Wasmuth 2025) and the characterization of patterns of variation across the genome, including structural variants (SVs) (Amarasinghe *et al.* 2020), runs of homozygosity (Brejon Lamartinière *et al.* 2024), and recombination landscapes (Weissensteiner *et al.* 2017). New sequencing approaches such as Hi-C (high-throughput chromosome conformation capture; Belton *et al.* 2012) have been instrumental in improving the contiguity of genome assemblies by allowing sequencing reads to be assembled into phased haplotypes. These techniques have already been used to generate chromosomal reference genomes for several cultured and domesticated fungi (Morin *et al.* 2012; Engel *et al.* 2013; Yu *et al.* 2022; Ma *et al.* 2023). However, to date, chromosomal reference genomes have only been published for 2 EMF, *Tricholoma matsutake* and *Suillus bovinus* (Kurokochi *et al.* 2023; Zhang *et al.* 2024).

Generating and analyzing long contiguous DNA sequences may represent the next frontier in genomic studies of EMF, as multiple recent studies of cultured and domesticated fungi have uncovered genomic structural variation both among and within dikaryons that may play a role in fungal evolution. For example, Sperschneider *et al.* (2023) used phased chromosomal assemblies of the arbuscular mycorrhizal fungus *Rhizophagus irregularis* to demonstrate that separate heterokaryon haplotypes are distinct functional and regulatory units that can independently modulate the expression of host plant genes. Similarly, Borgognone *et al.* (2018) showed that DNA methylation in the saprotrophic genus *Pleurotus* can be haplotype-specific and tends to be higher around transposable elements (TEs), where it reduces potentially maladaptive gene expression. As many EMF also have a heterokaryotic life stage, haplotype-resolved chromosomal EMF reference genomes are likely to become important resources for understanding the biology of this important group of fungi.

*Boletus edulis* Bull., known variously as the king Bolete, Penny Bun, cèpe de Bordeaux, Steinpilz or porcino, is one of the most charismatic and economically important EMF species worldwide. While the majority of commonly found EMF associate with just 1 or 2 host plant genera (Voller *et al.* 2024), *B. edulis* forms mutualistic associations with diverse plant genera, from the most dominant forest trees of the northern hemisphere (*Fagus*, *Quercus*, *Pinus*, *Picea*, *Betula*, *Castanea*, and *Pseudotsuga*) to alpine miniature shrubs (Treindl and Leuchtmann 2019). It also has a broad geographical distribution spanning Eurasia and North America. For these reasons, *B. edulis* has recently emerged as a promising model system for studying the ecology and evolution of EMF (Hoffman *et al.* 2020; Tremble *et al.* 2020; Tremble, Brejon Lamartinière, *et al.* 2023; Tremble, Hoffman, *et al.* 2023; Brejon Lamartinière *et al.* 2024).

The first global-scale population genomic study of *B. edulis* found evidence for six distinct lineages that diverged from one another between 1.6 and 2.7 million years ago (Tremble, Hoffman, *et al.* 2023). Reference genomes have already been generated for all of these lineages (Tremble, Brejon Lamartinière, *et al.* 2023), but they vary considerably in contiguity and completeness, and none of them are haplotype-resolved or assembled to the chromosomal level. The most contiguous of these reference genomes, which comprises a total of 38 scaffolds with an N50 of 2.5Mbp, is available for the Alaska lineage, while the least contiguous reference genome, which comprises 488 scaffolds with an N50 of 0.17 Mbp, is available for the European lineage. Moreover, these reference genomes were generated using haplotype-unaware methods, which limits the detection of SVs, especially in highly repetitive genomes (Ebert *et al.* 2021).

Within the *B. edulis* complex, the European lineage exhibits the widest geographical and ecological distribution, ranging from Mediterranean grasslands to the Scandinavian tundra. This lineage is also associated with the greatest diversity of host species (MyCoPortal, http://www.mycoportal.org/portal/index.php), which is reflected by its expanded symbiosis-related gene repertoire (Tremble, Brejon Lamartinière, *et al.* 2023). Developing a chromosomal reference genome for this lineage would allow for a more in-depth exploration of population structure, local adaptation, and host specialization, not only across Eurasia, but also on a broader scale. This is particularly important in the context of climate change, as many forests of the northern hemisphere are being strongly impacted by warmer and drier conditions (Gazol *et al.* 2017). A deeper understanding of forest adaptation is essential and this must include EMF, which play a vital role in helping trees cope with climate-induced stress.

In this study, we combined highly accurate long-read Pacbio HiFi sequencing with Hi-C to generate a haplotype-resolved chromosomal genome assembly for the European *B. edulis* lineage. We predicted key genomic features including mating loci, carbohydrate-active enzymes (CAZymes), TEs, and overall gene content. Additionally, we investigated structural and gene copy variation within the reference individual and explored longer-term patterns of synteny with *S. bovinus (*Zhang *et al.* 2024*)*. To further contextualize the reference genome, we mapped short-read data from 53 European samples to explore patterns of population genetic structure and diversity.

## Materials and methods

### Sporocarp tissue sampling

A total of 15 g of tissue from the inner cap flesh of a *B. edulis* sporocarp was collected from a *Fagus* woodland in Bielefeld, Germany. It was sequentially frozen at 4°C for 30 min and then at −20°C for 30 min, before being stored at −80°C. DNA isolation, library preparation, sequencing, and genome component prediction were performed by Biomarker Technologies GmbH as described below. In addition, tissue from the same individual was dried and archived in the Senckenberg Museum of Natural History, Görlitz, under the reference GLM-F139661.

### Genomic DNA isolation and sequencing

DNA was extracted from the sporocarp tissue using a QIAGEN Genomic-tip 20G kit. To generate a highly contiguous assembly, long-read sequencing was conducted as follows. Libraries were constructed according to PacBio standard protocol and sequenced on a PacBio Sequel II platform by BMKgene. The resulting raw circular consensus sequences were quality-filtered using smrtlink v12 with the parameters –min-passes 5 –min-rq 0.9, assembled into scaffolds using Hifiasm v0.12 (Cheng *et al.* 2021), and corrected using Pilon v1.17 (Walker *et al.* 2014).

Hi-C libraries were constructed using the Illumina mate-pair kit and sequenced on an Illumina NovaSeq X plus platform. The resulting paired-end Hi-C reads were filtered using HiC-Pro v2.10.0 (Servant *et al.* 2015), separated into haplotypes through integration with long reads using Hifiasm v0.12, and aligned to the long-read assembly using bwa v0.7.10 (Li and Durbin 2009). The contigs were then clustered, ordered, and oriented using Lachesis (Burton *et al.* 2013). To visualize chromatin interactions and genome contiguity, we generated a chromatin contact heat map using the command-line version of Juicer v1.6 (Durand *et al.* 2016).

### Preliminary genome annotation

A repeat database was constructed using a combination of LTR_FINDER v1.05 (Xu and Wang 2007), MITE-Hunter (Han and Wessler 2010), RepeatScout v1.0.5 (Price *et al.* 2005), and PILER-DF v2.4 (Edgar and Myers 2005). The database was sorted with PASTEClassifier (Hoede *et al.* 2014) and merged with the Repbase database (Bao *et al.* 2015). RepeatMasker v4.0.6 (Smit et al. (2013-2015)) was then used to predict repeat elements in the fungal genome based on this combined database. Gene prediction was performed through both de novo and homology-based approaches. The de novo prediction utilized Genscan (Burge and Karlin 1997), Augustus v2.4 (Stanke *et al.* 2004), GlimmerHMM v3.0.4 (Majoros *et al.* 2004), GeneID v1.4 (Alioto *et al.* 2018), and SNAP (version 2006-07-28) (Johnson *et al.* 2008). For homology-based prediction, GeMoMa v1.3.1 (Keilwagen *et al.* 2019) was used to identify homologous protein-coding genes. The results from both approaches were integrated using EvidenceModeler v1.1.1 (Haas *et al.* 2008). Noncoding RNA was predicted using tRNAscan-SE -version 2.0 (Chan and Lowe 2019) for tRNA and Infernal 1.1 (Nawrocki and Eddy 2013) for other noncoding RNAs. Pseudogenes were identified by aligning homologous genes from the predicted protein list and the Swiss-Prot database (Bairoch and Apweiler 2000) to the genome using GenBlastA v1.0.4 (She *et al.* 2009), followed by the detection of early termination and frameshift mutations with GeneWise (Birney *et al.* 2004). Gene clusters were identified using antiSMASH v6.0.0 (Medema *et al.* 2011). CAZymes encoding genes were annotated using hmmer -v3.4 (Finn *et al.* 2011) based on the CAZy database (Cantarel *et al.* 2009). Loci encoding effector proteins were predicted using EffectorP v3.0 (Sperschneider *et al.* 2016). Approximate centromere locations were identified visually based on Hi-C interactions. AT content at these locations was inspected in R using 1-kb sliding windows.

### Haplotype comparisons and evolutionary context

To investigate the quality of the alternative haplotypes, we identified the positions of conserved Benchmarking Universal Single-Copy Orthologs (BUSCO) genes from the basidiomycota_obd10 database using BUSCO v5.7.1 (Simão *et al.* 2015) separately for each haplotype. We also ran gene and TE prediction on each haplotype using Funnanotate v1.8.17 (Palmer and Stajich (2023)) and EDTA v2.2.0 (Ou *et al.* 2019), respectively. To evaluate mapping coverage, we aligned sequencing reads from 53 European *B. edulis* samples to both haplotypes using the mem2 algorithm from bwa (https://github.com/bwa-mem2/bwa-mem2) with the default parameters. These sequencing data included previously published Illumina MiSeq, HiSeq, and NovaSeq data from 49 samples (Tremble, Brejon Lamartinière, *et al.* 2023) as well as 4 additional samples from Bielefeld, Germany, that were 150 bp PE sequenced to 30 × coverage on a BGI T7 platform. To compute the average depth of coverage per position, we used a 500-kbp sliding window with a 50-kbp step, combining the VCFtools command –site-mean-depth (Danecek *et al.* 2011) and the GenomicRanges Grange function in R (Lawrence *et al.* 2013).

After characterizing and comparing the 2 haplotypes, as described in the results, we selected haplotype 2 for all subsequent analyses. The mating locus MATa identified by (Tremble, Brejon Lamartinière, *et al.* 2023) and the annotated STE3 genes from the MATb locus in the *B. edulis* bed1 genome accessed from JGI MycoCosm (Miyauchi *et al.* 2020) were located in the reference genome using BLAST v2.2.31 (Altschul *et al.* 1990). We also checked for the presence of telomeric sequences and located them in the genome using the Telomere Identification Toolkit TIDK (Brown *et al.* 2023). Finally, we analyzed patterns of synteny between the *B. edulis* reference genome and the chromosomal assembly of *S. bovinus* (Zhang *et al.* 2024) through the identification and mapping of putatively identical Basidiomycota BUSCO genes.

### European population structure and genetic diversity

As a first application of this newly generated resource, we analyzed patterns of population genetic structure and genome-wide diversity within the European *B. edulis* lineage. After mapping short-read data from 53 genets to haplotype 2 of the reference genome following the workflow described above, variants were called using a 3-step approach implemented in GATK v4.4.0 (McKenna *et al.* 2010). First, variants were called with HaplotypeCaller, then, the files were aggregated into a single database using GenomicsDBImport, and finally, an all-site unfiltered multi-sample VCF file was generated with GenotypeGVCF. This file was then filtered for single-nucleotide polymorphisms (SNPs) with a minor allele frequency (MAF) ≥ 5%, mapping quality ≥ 15, missing genotypes ≤ 20%, a minimum depth of coverage of 5, and a maximum depth of coverage of 100 using VCFtools. We then computed a principal component analysis (PCA) of the data using pcadapt (Luu *et al.* 2017) on the filtered VCF. In addition, we calculated nucleotide diversity (π) in windows of 10 kb with a step size of 1 kb across the genome. This analysis was conducted separately for individuals from different geographical regions with a sample size greater than 5 using the VCFtools –window-pi and –window-pi-step commands. The all-site VCF included invariant loci, which were pruned as described previously, while only excluding the MAF filter.

## Results and discussion

### Genome assembly quality

**Fig. 1.**
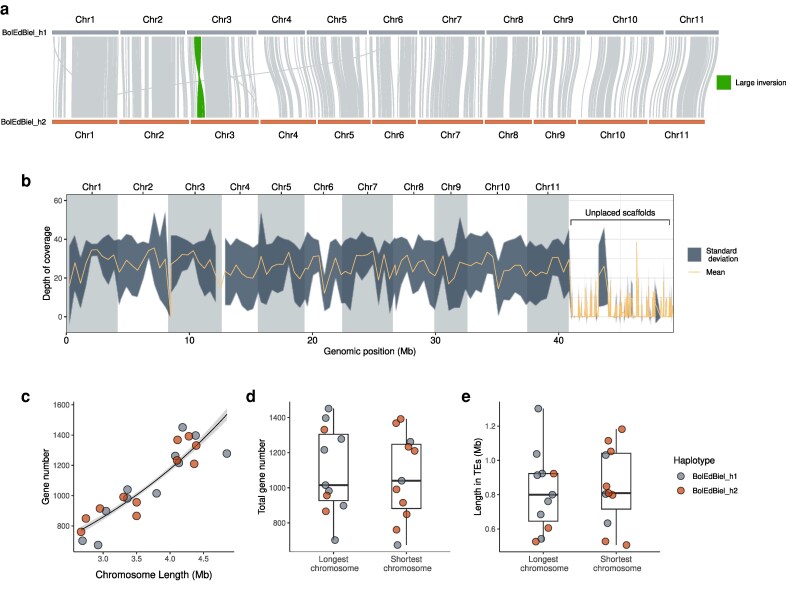
Comparison of the 2 *B. edulis* haplotype assemblies. a) Patterns of synteny between the 2 haplotypes of each pair of homologous chromosomes. To facilitate visualization, we reversed and complemented the alignments of chromosomes 5, 6, 7, and 9 from haplotype 1 and highlighted the inversion on chromosome 3 in green. b) Depth of mapping coverage of whole-genome resequencing data from 53 genets from the European *B. edulis* linage to haplotype 1. The orange line represents the mean mapping coverage, with the dark gray shaded region representing the 95% confidence interval. The chromosomes are represented by alternating gray and white blocks. c) The relationship between chromosome length and gene number. The black line represents the fit of a Poisson regression with the gray shaded region representing the SE. d) and e) show comparisons of gene number counts and TE content between the longest and shortest haplotypes, respectively. In a), c), d), and e), haplotypes 1 and 2 are color-coded in gray and orange, respectively.

The PacBio-generated scaffolds achieved a 100% Hi-C anchoring rate for both haplotypes, resulting in 2 chromosome-level assemblies consisting of 11 pseudo-chromosomes each (“BolEdBiel_h1” and “BolEdBiel_h2,” respectively, Table 1, Fig. 1a, and Supplementary Fig. 1 and 2; see Supplementary Tables 1 and 2 for details). The high quality of these assemblies is evident from their excellent chromosome contiguity, low numbers of gaps per chromosome (Table 2), and high BUSCO scores, which exceeded 96.6% for both haplotypes, with the score being slightly higher for haplotype 2. Genome sizes for the 2 haplotypes were 53 and 41 Mbp, respectively, with both exhibiting a GC content of 53.7%. However, the scaffold N50 was slightly lower for haplotype 1 (3.8 Mbp compared with 4.1 Mbp for haplotype 2). This discrepancy is partly due to the fact that over 15% of the total length of haplotype 1 comprised unanchored scaffolds. When mapping short-read sequencing data from 53 European individuals to this haplotype, coverage for all but one unplaced scaffold was very low (mean = 2.2 vs 25.9, respectively, Fig. 1b), reflecting their high content of repetitive DNA.

**Table 1.** Quality metrics for the *B. edulis* haplotype assemblies.

Haplotype	Length (bp)	Chromosomes	Scaffolds	Contigs	Contig N50 (bp)	Scaffold N50 (bp)	*n* BUSCOs (%)	%GC content	Total length in transposable elements	Total length in repetitive DNA
BolEdBiel_h1	53,135,892	11	311	321	3,363,843	3,798,680	C: 1,704 (96.6%) S: 1,630 (92.4%) D: 74 (4.2%) F: 19 (1.1%) M: 41 (2.3%)	53.7	9,434,133	3,330,036
BolEdBiel_h2	41,815,337	11	73	90	2,952,305	4,104,162	C: 1,709 (96.8%) S: 1,643 (93.1%) D: 66 (3.7%) F: 17 (1%) M: 38 (2.2%)	53.7	8,893,489	3,536,600

C, complete; S, complete and single copy; D, complete and duplicated; F, fragmented; M, missing.

**Table 2.** Length and number of gaps per chromosome from each haplotype.

Chromosome	BolEdBiel_h1 length	BolEdBiel_h1 gaps	BolEdBiel_h2 length	BolEdBiel_h2 gaps
1	4,187,054	4	4,111,618	0
2	4,077,455	1	4,390,755	1
3	4,381,500	0	4,281,452	1
4	2,924,582	0	3,539,504	5
5	3,798,680	0	3,304,873	0
6	3,044,205	1	2,742,422	2
7	4,131,713	1	4,104,162	0
8	3,361,260	0	2,952,305	0
9	2,691,760	3	2,670,009	3
10	4,848,437	0	4,360,388	4
11	3,363,843	0	3,500,508	1

We found a strong positive association between chromosome length and gene number (*z* = 30.39, *P* < 0.001, Fig. 1c). However, this relationship did not hold when comparing homologous chromosomes. In most cases, haplotype 1 was longer than haplotype 2 (Fig. 1a and d), yet there were no significant differences between homologous chromosomes in terms of gene count (Fig. 1d) and the abundance of TEs (Fig. 1e). Based on these results and the slightly better quality metrics for haplotype 2, including higher scaffold N50 and BUSCO scores, along with the low coverage of haplotype 1-specific contigs, we selected BolEdBiel_h2 as the preferred reference genome for the European lineage of *B. edulis*. Nonetheless, both haplotypes have excellent quality metrics and, given that there is no linkage across chromosomes, the choice of haplotype is somewhat arbitrary. Therefore, both assemblies could potentially be used for research in this species, particularly for pan-genomic or structural variation analyses.

The synteny analysis also uncovered a 0.4-Mbp inversion on chromosome 3 (Fig. 1a). We confirmed this inversion by evaluating PacBio sequence mapping rates using the integrative genomics viewer (Thorvaldsdóttir *et al.* 2012). Inversions can play important roles in evolution by limiting or suppressing local recombination, which helps to maintain genetic diversity and adaptive potential, and can also drive population divergence and speciation (Hoffmann and Rieseberg 2008). In extreme cases, inversions have been found to induce shifts from mutualism to pathogenesis (Somvanshi *et al.* 2012). Consequently, further research into this SV might help to illuminate the genetic mechanisms underlying the host adaptability and ecological flexibility of *B. edulis*.

### Genome content

**Fig. 2.**
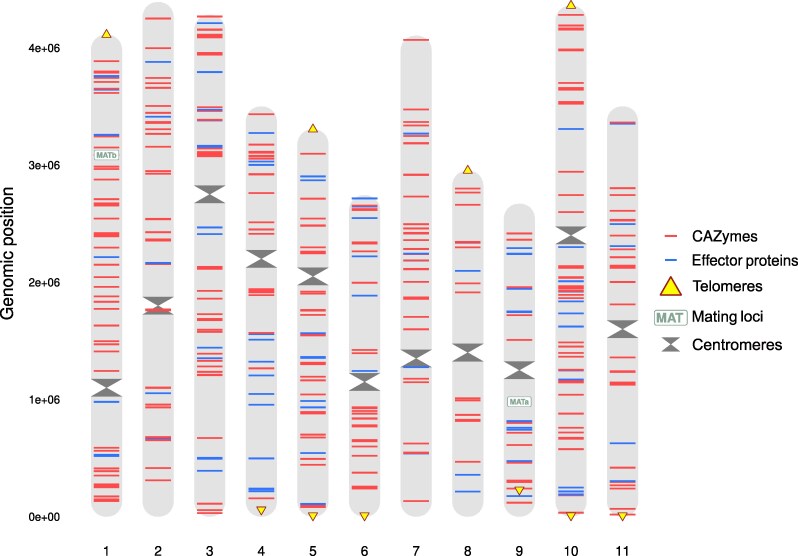
The distribution of key features in the *B. edulis* reference genome. Shown are the genomic locations (in haplotype 2, BolEdBiel_h2) of mating loci, genes encoding CAZymes and effector proteins, centromeres, and telomeres.

Focusing on BolEdBiel_h2, we de novo predicted a total of 15,406 genes with an average length of 1.7 kb, which together account for ∼53.4% of the total genome length (41.8 Mbp). Additionally, we identified 11,890 repetitive sequences, mostly comprising long terminal repeat retrotransposons (Supplementary Fig. 3), which represented 35.4% of the genome length in chromosomes. Ten telomeric repeats, (TTAGG)*n*, were identified in 8 of the 11 chromosomes. Near telomere-to-telomere assemblies were achieved for chromosomes 5 and 10, with the former being gapless (Fig. 2 and Table 2), which is a rarity in EMF. Telomeres, and chromosome ends in general, have been linked to several important biological processes. For example, in the fungal pathogen *Pyricularia*, telomere-adjacent regions are enriched for genes involved in host adaptation (Rahnama *et al.* 2021). Similar patterns might be expected in EMF. In addition, the centromeres were identified based on their elevated interaction frequencies within the 3-dimensional chromatin architecture (Mizuguchi *et al.* 2014) which was visible in the Hi-C contact data (Supplementary Figs. 1 and 2). We found that, for most of the chromosomes, the centromere region exhibited high AT content relative to the rest of the chromosome (Supplementary Fig. 2). A similar pattern has been reported in other fungi, including the yeasts *Candida glabrata* and *Saccharomyces cerevisiae, both of which possess centromeric DNA elements with an AT content exceeding 85%* (Roy and Sanyal 2011).

We sought to identify the genomic locations of genes encoding carbohydrate-active enzymes (CAZymes), which have evolved in fungi to degrade carbohydrates such as lignin and cellulose. While typically associated with saprotrophism, CAZymes are also present to a lesser extent in EMF where they may support nutrient acquisition (Gong *et al.* 2023). We annotated 413 genes as encoding CAZymes, representing 2.7% of the total gene content. This is comparable to the 2.2% reported for *S. bovinus*, another EMF species (Zhang *et al.* 2024). These genes were unevenly distributed across chromosomes, with chromosome 9 carrying only 15 CAZymes compared with 52 on chromosome 1 (Fig. 2). We also identified genomic regions where multiple CAZymes clustered together. For example, at position 14,673,340 in the genome (located on chromosome 4), 4 CAZymes were found within a 54-kbp region (Fig. 2). These clusters may represent remnants of an ancestral presymbiotic lifestyle, as similar gene arrangements have been observed in wood-decaying fungi (Li *et al.* 2017). Further exploration of these CAZyme clusters might therefore provide insights into how EMF adapt to diverse environmental conditions.

Effector proteins are secreted by pathogenic fungi to manipulate host defenses during colonization (Li *et al.* 2024). They have also been shown to play a similar role during the early establishment of mycorrhizal symbioses, facilitating fungal colonization of host roots (Li *et al.* 2024). However, this process remains poorly understood, and only a small number of EMF effectors have been linked to plant gene expression pathways (Plett *et al.* 2011; Daguerre *et al.* 2020). Identifying these genes in the reference genome is therefore an important first step toward understanding their function and regulation in this species. We identified a total of 116 genes putatively encoding effector proteins. In pathogenic fungi, effector genes are often clustered in genomic regions experiencing rapid evolution, such as near telomeres (Zaccaron *et al.* 2022). However, the flanking regions of the 10 telomeric sequences identified here did not appear to be enriched for genes encoding effector proteins (Fig. 2).

Mating type (MAT) loci play a major role in determining sexual compatibility and reproduction in fungi (Coelho *et al.* 2017). In this tetrapolar species, we identified both mating loci: MATa on chromosome 9 and MATb on chromosome 1 (Fig. 2). Tetrapolar species are generally considered less prone to selfing than bipolar species. In line with this, a recent study of *B. edulis* found no evidence of recent inbreeding (Brejon Lamartinière *et al.* 2024), despite the presence of locally elevated relatedness (Hoffman *et al.* 2020). This suggests that the mating loci may contribute toward the maintenance of high levels of heterozygosity and genetic diversity within *B. edulis* populations. Future studies could use pedigrees to model the involvement of MAT loci in mating outcomes and inbreeding/outbreeding dynamics.

### Patterns of synteny

**Fig. 3.**
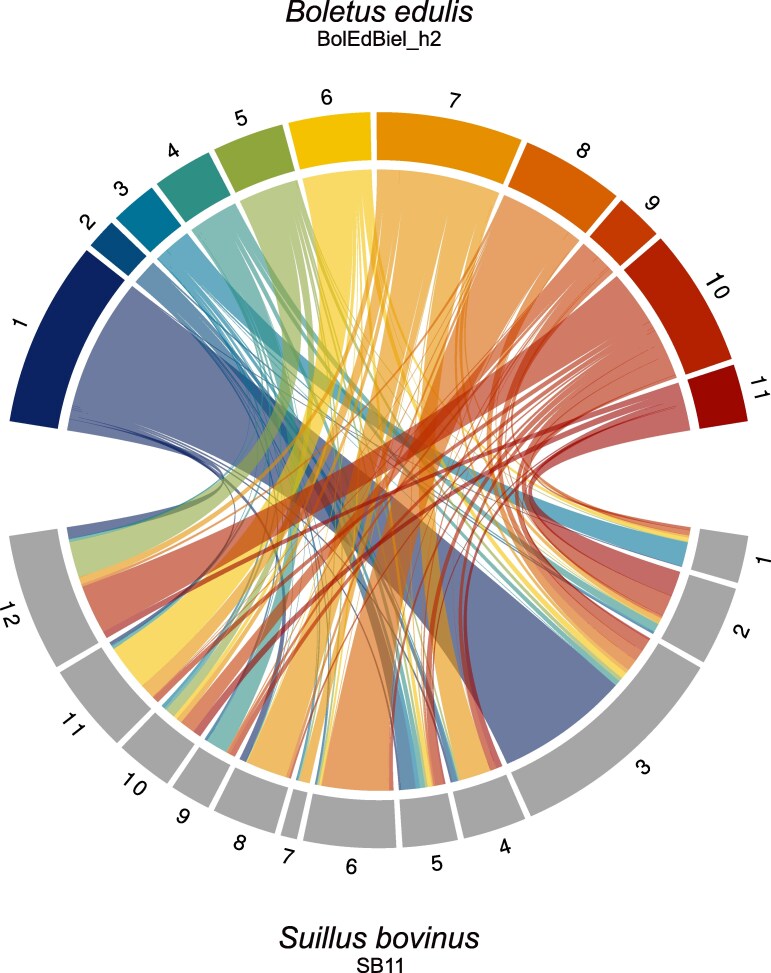
Patterns of chromosomal synteny between *B. edulis* and *S. bovinus*. The Circos plot depicts alignments of BUSCO genes from haplotype 2 of *B. edulis* (top) to the reference genome of *S. bovinus* (below). The *B. edulis* chromosomes are shown in color, and the *S. bovinus* chromosomes are shown in gray.

To provide phylogenetic and evolutionary context, we investigated patterns of synteny between *B. edulis* and *S. bovinus* based on shared BUSCO genes. While both species belong to the order Boletales, *S. bovinus* is part of the family Suillaceae, which diverged from Boletaceae ∼115 million years ago (Wu *et al.* 2022). Despite sharing an ectomycorrhizal lifestyle with *B. edulis*, *S. bovinus* has a more restricted host range, primarily associating with Pinaceae (Zhang *et al.* 2024). Surprisingly, we observed high levels of synteny between these species (Fig. 3), despite their considerable phylogenetic distance, differences in chromosome numbers, and the structural and size variation present between homologous *B. edulis* haplotypes (Supplementary Table 1). Notably, large BUSCO gene segments on chromosomes 1 and 8 of *B. edulis* showed structural conservation with chromosomes 3 and 6 respectively of *S. bovinus* (Fig. 3). Generating additional chromosomal reference genomes for other EMF species would provide deeper insights into genome evolution through comparative chromosomal synteny analyses across multiple species.

### Population structure and genetic diversity

**Fig. 4.**
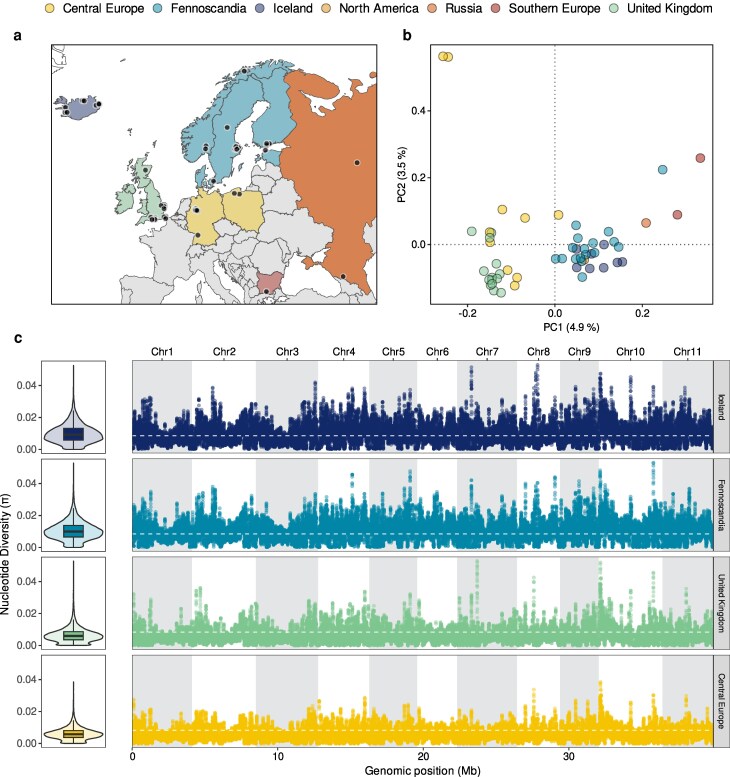
Population structure and genetic diversity of the European lineage of *B. edulis*. a) Sampling locations of 53 genets, color-coded by geographical region. b) Scatter plots of individual variation in principal component (PC) scores derived from PCA of the genomic data. The amounts of variation explained by each PC are given as percentages on the axis labels, and the samples are color-coded as shown in a). c) Nucleotide diversity (π) calculated in 10-kb sliding windows across the genome for every population. The left panels show violin plots of the kernel densities of π together with standard Tukey boxplots (center line = median, bounds of the box = 25th and 75% percentiles, upper and lower whiskers = largest and smallest values but no further than 1.5 * inter-quartile range from the hinge). The right panels show π values for each window across the 11 chromosomes. The dashed white lines represent lineage-specific genome-wide average π values.

We analyzed short-read data from 53 genets (Fig. 4a, Supplementary Table 3, Tremble, Hoffman, *et al.* 2023; Brejon Lamartinière *et al.* 2024, and this manuscript) to genotype 937,328 SNPs for the assessment of population structure and genetic diversity within the European *B. edulis* lineage. Two main clusters were resolved in the PCA, with samples from the United Kingdom and Central Europe separating from samples from Iceland and Fennoscandia along the first axis (Fig. 4b). Notably, a single genet sampled in North America, which was assigned to the European lineage by Tremble, Hoffman, *et al.* (2023), clustered in the PCA alongside the Fennoscandian and Icelandic samples (Fig. 4b). Although we cannot be certain, this suggests that this individual may have been introduced to America from a tree plantation originating in northern Europe.

Genetic diversity was not evenly distributed among the genetic clusters (Fig. 4b). Populations at higher latitudes, specifically those in Iceland and Fennoscandia, exhibited higher genome-wide nucleotide diversity (π) than populations from the United Kingdom and Central Europe. This observation aligns with our previous finding of a negative (but nonsignificant) association between latitude and genomic inbreeding within the European *B. edulis* lineage (Brejon Lamartinière *et al.* 2024). Several factors may contribute to this pattern, including greater habitat availability and continuity in the north, where much of Iceland and Fennoscandia remains covered by forests or tundra. Additionally, differences in the amount of gene flow with the neighboring “AK’ lineage, which spans Alaska and Siberia, may also play a role (Tremble, Brejon Lamartinière *et al.* 2023). Above and beyond this pattern, we also identified regions of elevated π within the *B. edulis* genome. Some of these regions were shared among the clusters, such as a prominent peak near the beginning of chromosome 10, while others were not universally shared, appearing only on specific chromosomes (Fig. 4c). These regions could represent areas of structural variation, they may be under balancing selection, or they might experience locally elevated recombination. This highlights the potential of the European lineage of *B. edulis* as a model for studying these diverse evolutionary processes.

### Conclusion

Population genomic studies of EMF hold great potential for advancing our understanding of these ecologically important organisms. However, the contiguity of reference genomes continues to be a limiting factor. This study presents one of the first haplotype-resolved chromosomal reference assemblies for a dikaryotic EMF, achieving near telomere-to-telomere coverage across multiple chromosomes. Using chromatin conformation capture, we successfully anchored PacBio long reads to chromosomes for each haplotype. This approach revealed structural variation within homologous chromosomes of the reference individual and identified key areas for future research. Specifically, we identified mating loci, CAZymes and effector proteins, discovered a 0.4-Mb inversion on chromosome 3, investigated the population genetic structure of the European lineage, and discovered several genomic regions with locally elevated nucleotide diversity. We anticipate that this new resource will facilitate future discoveries in this enigmatic and ecologically important fungus.

## Supplementary Material

jkaf069_Supplementary_Data

## Data Availability

The code, reference assembly, the Hi-C and Pacbio raw reads generated in this study, and the annotation are available at Zenodo (DOI: 10.5281/zenodo.14311982). The genome assembly and the newly generated short reads are deposited at NCBI under the BioProject PRJNA1187522. All sequences used in this study are publicly available, and their corresponding accession numbers are listed in Supplementary Table 3. Supplemental material available at G3 online.
