# Supplementary material for: A haplotype-resolved chromosomal reference genome for the porcini mushroom Boletus edulis
Source: G3 (Bethesda). 2025 Apr 24;15(6):jkaf069. doi: 10.1093/g3journal/jkaf069 (PMC12134998; doi:10.1093/g3journal/jkaf069)

Supplementary document for:

A haplotype-resolved chromosomal reference genome for the porcini mushroom *Boletus edulis*

Etienne Brejon Lamartinière, Keaton Tremble, Bryn T.M. Dentinger, Kanchon K. Dasmahapatra, Joseph I. Hoffman

Table S1: Hi-Fi read quality statistics

Read number	Read per base	Read length N50	Average read length	Maximum read length
959,239	8.227	9,657	8,577	42,710

Table S2. Hi-Fi read length distribution.

Length (bp)	Read number	Total length (bp)	Average length
1 to 2000	2,016	3,592,812	1,782.15
2000 to 4000	78,600	250,913,855	3,192.29
4000 to 6000	140,464	713,673,492	5,080.83
6000 to 8000	212,617	1,497,550,479	7,043.42
8000 to 10000	221,633	1,988,321,424	8,971.23
10000 to 12000	157,492	1,719,712,753	10,919.37
12000 to 14000	87,073	1,122,216,264	12,888.22
14000 to 16000	40,479	601,188,264	14,851.86
16000 to 18000	14,188	238,322,175	16,797.45
Greater than 18000	4,677	91,557,603	19,576.14

Table S3 Metadata of the sequences used in this study.

SampleID	Ascencion ID	BioSample ID	lat	lon	State /country	Collection year	BioProject
60	60	SAMN21445538	56.98296	-3.38683	Scotland	2009	PRJNA1216376
242	242	SAMN21445524	53.95252	17.9191	Poland	2015	PRJNA1216376
244	244	SAMN21445525	53.89653	16.08535	Poland	2015	PRJNA1216376
B140	B140	SAMN21445544	52.3859	0.736607	England	2015	PRJNA1216376
B51	B51	SAMN21445545	52.39074	0.730854	England	2015	PRJNA1216376
B53	B53	SAMN21445546	52.3903	0.736675	England	2015	PRJNA1216376
BD572	K(M) 200472	SAMN21445549	41.50508	24.33587	Greece	2011	PRJNA1216376
BD591	K(M) 263350	SAMN21445550	51.06465	-0.09363	England	2014	PRJNA1216376
BD592	K(M) 263351	SAMN21445551	51.06348	-0.09509	England	2014	PRJNA1216376
BD593	K(M) 263352	SAMN21445552	51.06316	-0.99161	England	2014	PRJNA1216376
BD594	K(M) 263353	SAMN21445553	50.90453	-1.59178	England	2014	PRJNA1216376
BD595	K(M) 263354	SAMN21445554	50.90453	-1.59178	England	2014	PRJNA1216376
BD596	K(M) 263355	SAMN21445555	50.88668	-1.55498	England	2014	PRJNA1216376
BD598	K(M) 263357	SAMN21445557	50.88663	-1.5549	England	2014	PRJNA1216376
BD599	K(M) 263358	SAMN21445558	50.88667	-1.55497	England	2014	PRJNA1216376
Bi126	Bi126	SAMN21445559	52.06102	8.471382	Germany	2015	PRJNA1216376
Bi58	Bi58	SAMN21445560	52.0381	8.486884	Germany	2015	PRJNA1216376
Bi81	Bi81	SAMN21445561	52.0381	8.486884	Germany	2015	PRJNA1216376
C1411	C1411	SAMN21445564	52.46192	0.677378	England	2014	PRJNA1216376
CEP53	CEP53	SAMN21445568	52.36469	0.614834	England	2009	PRJNA1216376
C-F-109468	C-F-109468	SAMN21445562	55.75926	12.36562	Denmark	2018	PRJNA1216376
C-F-118985	C-F-118985	SAMN21445563	62.52604	15.62133	Sweden	1987	PRJNA1216376
F-75196	O-F-75196	SAMN21445587	60.2922	10.6895	Norway	2014	PRJNA1216376
F-75615	O-F-75195	SAMN21445588	59.73594	10.72811	Norway	2014	PRJNA1216376
FA-09455	FA-09455	SAMN38089244	65.7	-17.875	Iceland	1984	PRJNA1216376
FA-11071	FA-11071	SAMN38089245	65.261	-14.375	Iceland	1987	PRJNA1216376
FA-13148	FA-13148	SAMN38089246	65.127	-22.168	Iceland	1989	PRJNA1216376
FA-13860	FA-13860	SAMN38089247	65.243	-14.348	Iceland	1984	PRJNA1216376

FA-13861	FA-13861	SAMN38089248	66.061558`	-18.651804	Iceland	1980	PRJNA1216376
FA-16929	FA-16929	SAMN21445589	65.70826	-17.8866	Iceland	2002	PRJNA1216376
FA-18247	FA-18247	SAMN21445590	64.52307	-21.4344	Iceland	2005	PRJNA1216376
FA-18312	FA-18312	SAMN21445591	64.53241	-21.4371	Iceland	2005	PRJNA1216376
MIN-921562	MIN921562	SAMN21445614	41.95442	-78.742	Pennsylvania	2003	PRJNA1216376
MIN-921977	MIN921977	SAMN38089293	59.815762	17.533535	Sweden	2005	PRJNA1216376
MIN-921978	MIN921978	SAMN38089294	59.816496	17.53332	Sweden	2005	PRJNA1216376
MIN-921981	MIN921981	SAMN21445617	59.833	17.6	Swedenv.	2005	PRJNA1216376
MIN-921982	MIN921982	SAMN38089295	59.836801	17.624128	Sweden.	2005	PRJNA1216376
MIN-921983	MIN921983	SAMN38089296	59.854706	17.632778	Sweden.	2005	PRJNA1216376
MIN-921984	MIN921984	SAMN38089297	59.854706	17.632778	Sweden.	2005	PRJNA1216376
MIN-921988	MIN921988	SAMN21445619	59.83695	17.6231	Sweden	2005	PRJNA1216376
MIN-921989	MIN921989	SAMN47006298	59.839628	17.622458	Sweden	2005	PRJNA1216376
N1	N1	SAMN38089299	58.160865	45.471601	Russia	2004	PRJNA1216376
R029174	KR-M-0029174	SAMN21445630	48.95715	8.521229	Germany	2011	PRJNA1216376
TAAM-132367	TAAM132367	SAMN21445642	60.297	24.9628	Finland	1994	PRJNA1216376
TAAM-132806	TAAM132806	SAMN38089338	60.21275	24.97465	Finland	1992	PRJNA1216376
TAAM-132820	TAAM132820	SAMN38089340	60.30501	24.96475	Finland	1992	PRJNA1216376
TAAM-175085	TAAM175085	SAMN21445645	43.38729	41.70553	Russia	1999	PRJNA1216376
TAAM-185627	TAAM185627	SAMN21445646	59.58	24.505	Estonia	2003	PRJNA1216376
TR39322	TROM-39322	SAMN21445647	69.72064	19.26532	Norway	2001	PRJNA1216376
B17009	B17009	SAMN45203299	52	8.4	Germany	2017	PRJNA1187522
B17042	B17042	SAMN45203301	52	8.4	Germany	2017	PRJNA1187522
B17060	B17060	SAMN45203302	52	8.4	Germany	2017	PRJNA1187522
B17010	B17010	SAMN45203300	52	8.4	Germany	2017	PRJNA1187522
BolEdBiel	BolEdBiel_h2	SAMN44794632	52	8.4	Germany	2023	PRJNA1187522

Figure S1: Hi-C contact heat-map of all bins from all chromosomes. Each bin is colored according to the number of Hi-C interactions, in binary logarithm format, such that darker colors represent higher number of interactions.

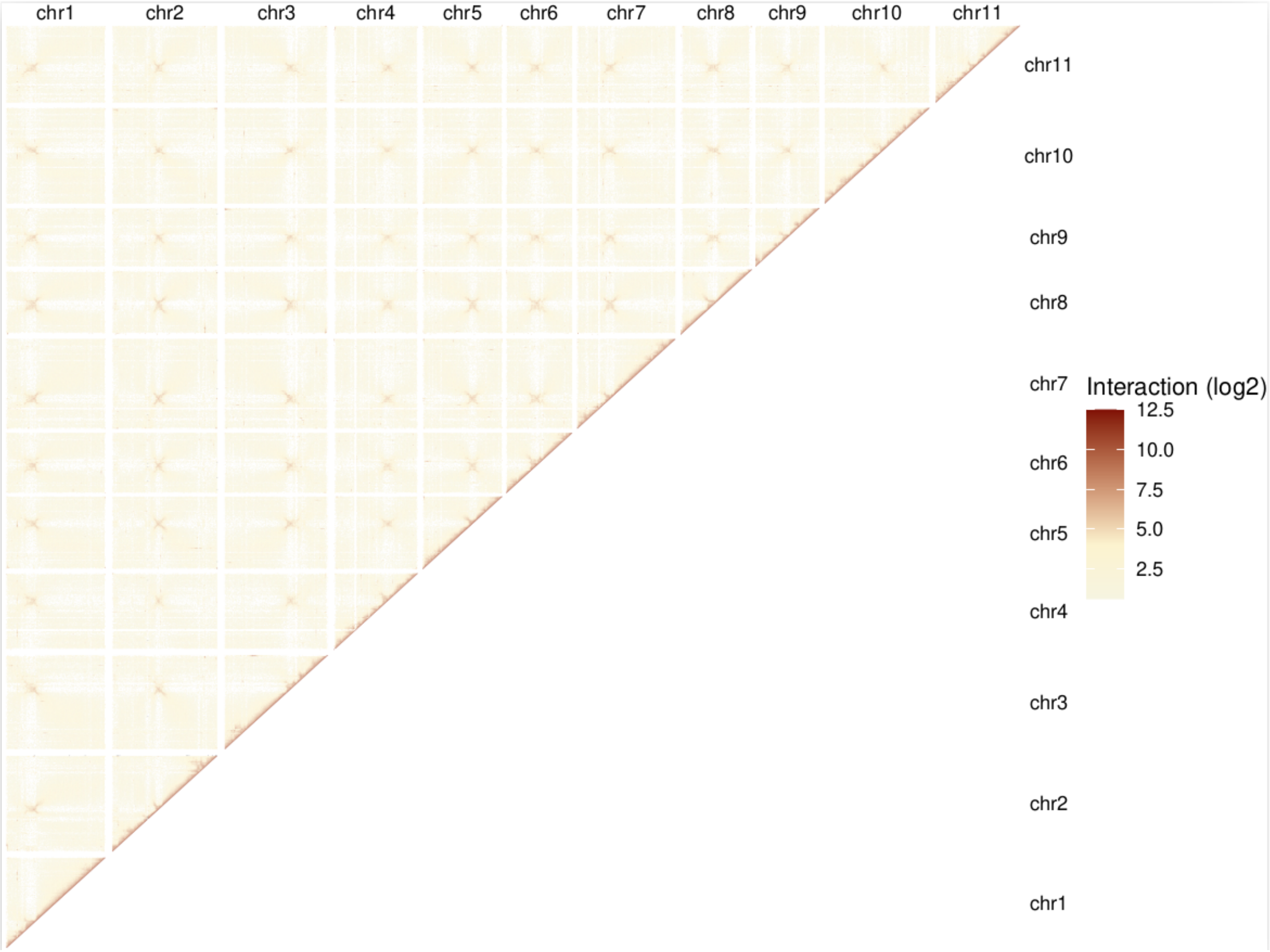

Figure S2: Chromosome wide AT content and highest number Hi-C interactions (in binary logarithm format) per chromosome. The red line represent the approximate location of the centromeres that can be inferred from the Hi-C data.

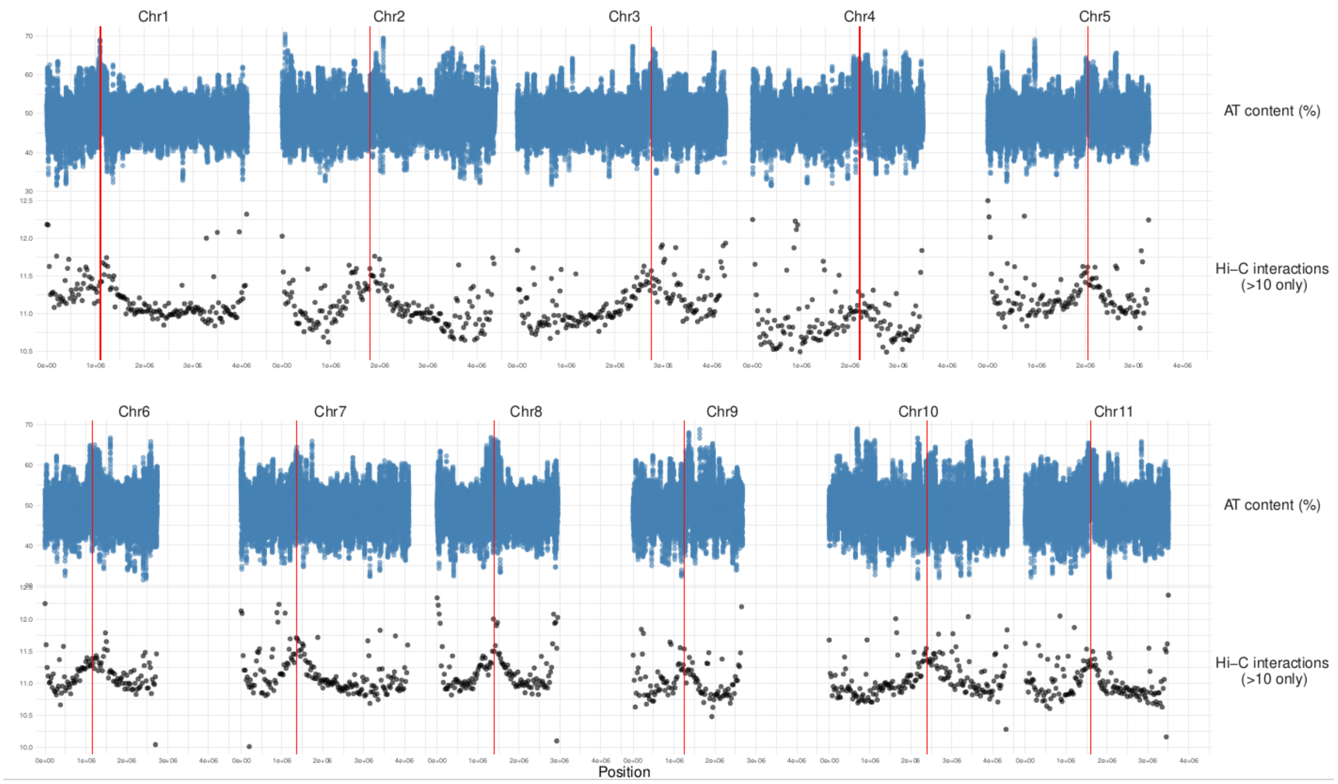

Figure S3: Breakdown of the categories of transposable elements found in both haplotypes, with their respective total length in bases.

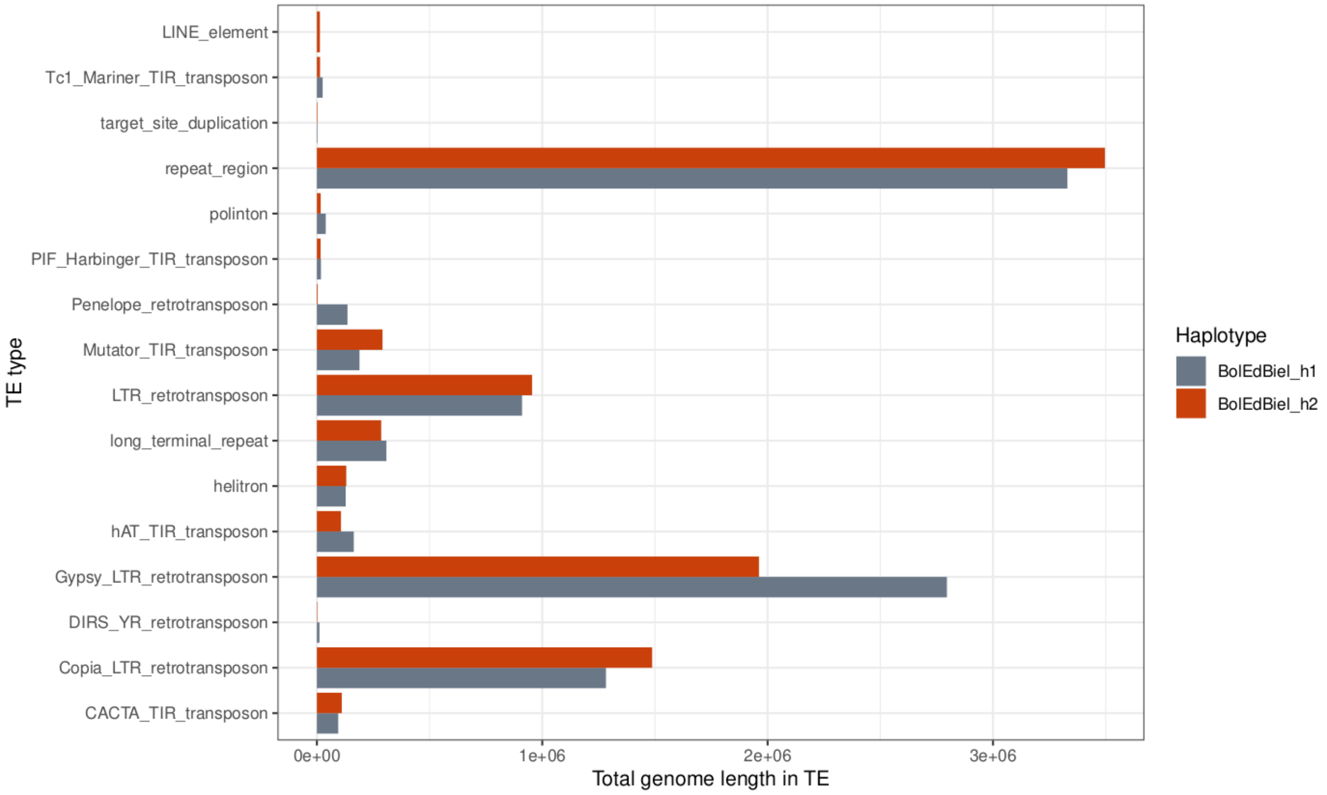

Supplement: jkaf069_Supplementary_Data [file jkaf069_supplementary_data.pdf]
